# Typological characteristics of interlanguage: Across L2 modalities and proficiency levels

**DOI:** 10.3389/fpsyg.2022.1071906

**Published:** 2023-01-02

**Authors:** Yuxin Hao, Xuan Xu, Xuelin Wang, Yanni Lin, Haitao Liu

**Affiliations:** ^1^Institute of Chinese Language and Culture Education, Huaqiao University, Xiamen, China; ^2^College of Chinese Language and Culture, Huaqiao University, Xiamen, China; ^3^College of Chinese Language and Culture, Jinan University, Guangzhou, China; ^4^College of Foreign Studies, Guangxi Normal University, Guilin, China; ^5^Center for Linguistics and Applied Linguistics, Guangdong University of Foreign Studies, Guangzhou, China; ^6^Department of Linguistics, Zhejiang University, Hangzhou, China

**Keywords:** interlanguage, typological characteristics, dependency direction, modalities, L2 proficiency

## Abstract

In recent years, quantitative methods have been increasingly used in interlanguage studies, but these studies have mostly focused on the micro level with an emphasis on certain syntactic structures, rather than the macro where interlanguage is perceived as a whole. There remains a paucity of quantitative studies on interlanguage from the typological perspective. With the majority of the studies focused on the written interlanguage, there is also a lack of sufficient research on its spoken modality. Based on a syntactically annotated corpus and using the quantitative linguistic metric of dependency direction, we have investigated the typological changes in the Chinese interlanguage in both written and spoken modalities. The findings are as follows: (1) the typological features of interlanguage vary across modalities at both macro and micro levels; (2) dependency direction is proved to be an inappropriate indicator to measure the general typological characteristics of interlanguage development due to its failure to reflect the changes in the spoken modality; (3) both macro and micro perspectives taken into consideration, typological errors in the interlanguage is more likely to occur in the spoken modality than in the written one, in which learners may be restricted by greater time pressure and cognitive load in utterance. These factors may affect the distribution of dependency direction in the oral modality, and may be the reason why it is not appropriate to use dependency direction as a measure of changes in mediated language typological features in the oral modality. It is expected that our study will bring insight into second language research with more objective and holistic evidence.

## Introduction

From the late 1960s to the early 1970s, Corder (1967, 1971), Selinker (1969, 1972) and Nemser (1971) put forward the theoretical hypothesis of “the language system of second language learners.” The theoretical hypothesis holds that learners' language system is an independent and complete system different from their mother tongue and target language, and has its own development laws. Nemser (1971) proposed and adopted the concept of “approximate system” to describe the language system of learners, and considered it as a continuum that gradually approaches the target language system and changes constantly. This language system produced by second language learners is called “interlanguage,” which is the natural language produced by second language learners when they acquire a new language (Richards et al., 2003). Since it is deemed as a natural language, interlanguage must also comply with the restrictions of linguistic universality (Tarone, 1979). In a word, interlanguage is an independent and progressive dynamic continuous system. There is a certain law of development in it, and it is also restricted by the general law of human language development.

In recent years, quantitative methods have been increasingly applied to the study of interlanguage and are considered as an effective means of validating interlanguage theory or studying the development of interlanguage. Rather, the plethora of interlanguage studies have focused on specific linguistic phenomena along various micro levels, such as L2 learners' lexicon (e.g., Yamashita and Jiang, 2010; Laufer and Waldman, 2011), semantic structures in L2 (e.g., Malt and Sloman, 2003; Tytus and Rundblad, 2017), the acquisition of specific syntactic structures (e.g., Izumi, 2003; Brandt, 2011; Yuan, 2015, 2017; Jach, 2018) and L2 learners' pragmatic acquisition (e.g., Zyzik, 2011; Hubers et al., 2020). However, empirical studies on the overall typological features and development process of interlanguage from a macroscopic view are rare, which may be mainly due to the lack of a mature syntactic analysis system for automatic computer processing of large-scale corpora in the past, and the failure to conduct exhaustive syntactic analysis of large-scale interlanguage corpora to extract continuous and data of overall syntactic features.

In order to conduct empirical research on interlanguage at a macro level and to understand the whole process of changes in the typological features of second languages, it is necessary to find an applicable syntactic theory. Quantitative linguistics, which uses mathematical and theoretical models to study the patterns of language structure, has developed considerably, in which syntactically annotated corpora as a tool for obtaining syntactic information can provide real and reliable data for syntactic studies (Meurers and Dickinson, 2017). Dependency grammar, which has long received much attention, has also taken on new life and recently become the dominant approach to annotate syntactic information in natural language processing (De Marneffe and Nivre, 2019). The research paradigm of quantitative linguistics is in line with the need to study the interlanguage system as a whole at a macro level (Liu and Huang, 2012). Based on the development of dependency grammars and natural language processing techniques, many syntactic parsers have been developed (Che et al., 2010; Chen and Manning, 2014), which can effectively be used to build a high-quality syntactic annotated corpus. Based on dependency grammar, a number of studies on the analysis of large-scale natural languages have made important breakthroughs (Liu, 2008; Hudson, 2010; Levshina, 2019; Yadav et al., 2020).

Dependency relations are the basis of dependency grammar. It refers to an asymmetric, directed, labeled relationship that exists between two syntactically related words (Hudson, 2007; Liu, 2009a; Tesnière, 2015). A dependency relation consists of three parts: the governor, the dependent, and the dependency relation label, the beginning of whose arrow is the governor and points to the dependent, and the label on the arc is the dependency relation label. Dependency direction, as an important concept of dependency grammar, can indicate whether the governor precedes or follows the dependent (Liu, 2010).

Greenberg (1966) pointed out that most of the commonalities of languages are related to the word order, which is a reliable typological indicator. Based on dependency syntactically annotated corpus, Liu (2010) conducted a quantitative study of 20 different languages and found that dependency direction reflects languages' overall word orders and can be used as a valid indicator of linguistic typology.^1^ Several subsequent studies have also confirmed the validity of the above approach (Liu and Xu, 2012; Jiang and Liu, 2015; Gerdes et al., 2019; Futrell et al., 2020; Hao et al., 2021). Jiang et al. (2019) conducted a study based on the writing corpus of eight consecutive grades of CESL learners (Chinese native speaking English second language learners), and the results showed that dependency direction can be a good measure of CESL learners' language proficiency and can be used as an indicator of the development of English interlanguage typological features. Hao et al. (2021), by using dependency direction as an indicator, studied the changes in the typological characteristics of ECSL learners (English native speaking Chinese second language learners) and JCSL learners (Japanese native speaking Chinese second language learners). They have found that dependency direction can be used as an indicator of the development of interlanguage typology other than English, providing strong evidence for its generality. However, the above studies have only focused on the written modality, and the application of dependency direction in the spoken modality of interlanguage has not received much attention.

Modality refers to a wide range of modes of communication (e.g. computer-mediated communication, face-to-face communication, sign language, etc.) and different stages of language processing (i.e., input, and output), but the scope of our study is limited to the two modalities of spoken and written languages in the output stage. This classification of spoken vs. written language is generally based on medium. For example, spoken language is oral language, involving the “mouth” and “ears”; written language is related to writing and involves mainly the “hands” and “eyes.” However, classifying language solely from the perspective of the medium may result in overlooking specific linguistic features. For example, a formal speech is oral in the medium, but its linguistic features are closer to written language than to the ordinary conversational genre, because the material for a formal speech is prepared in writing. As Ochs (1979) points out, spoken corpora can be classified as planned and unplanned according to the relative degree of prior thought and organizational effort put into them, with the unplanned spoken features being closer to the typical spoken language. To avoid this problem, Michael and Ronald (1994) proposed that corpora should be classified in terms of both medium and mode of expression, where the latter is the choice of linguistic features. Therefore, in order to ensure the rigor of the discussion and the accuracy of the results, this study only discusses spoken and written languages with typical linguistic characteristics.

Spoken and written languages have different cognitive processes and production patterns. Levelt (1989)'s speech production model consists of three components: the Conceptualizer, the Formulator, and the Articulator. The Conceptualizer works at the concept formation stage, where the speaker determines the intention to speak and the concept to be expressed based on prior knowledge (Towell et al., 1996). This preverbal message is transformed into a discourse plan through the phonemic encoding part of the Formulator, and finally into the Articulator. Scholars have made systematic studies on sound change as a result of interlanguage speakers (Chirkova and Gong, 2014). Hayes and Flower (1980) decomposed the written production model into three sub-processes, including planning, translating, and reviewing. Planning involves generating messages, setting composition goals, and organizing the information extracted from memory. Through translating, the ideas generated by the plan are converted into written texts. Reviewing text production involves reading texts and detecting errors or problems either in the texts or in the plans for texts. Thus, whereas both speaking and writing involve turning ideas into linguistic representation through hierarchically ordered processes, spoken language is a reflection of the process of language construction, whereas written language is a revised and polished product (Hayes and Flower, 1980; Halliday, 1989; Levelt, 1989; Kellogg, 2001; Cutting, 2011). In addition to this, the time pressure and cognitive burden of outputting oral language are heavier than those of written (Grabowski, 2007). Previous studies have found differences in the syntactic performance across spoken and written modalities in both first language systems and interlanguage systems (Biber, 1988; Kormos, 2014; Biber et al., 2016; Zalbidea, 2017; Cho, 2018).

Then, can the dependency direction be used as an indicator of the development of the typological features of the spoken modality of the interlanguage? Do such developmental features show differential characteristics in spoken and written modalities? Such studies are rare and only involve studies of native speaker corpora. Based on the dependency treebanks of five different genres of Chinese, Liu (2009b) found that the head-final (HF) dependency percentages in the CUCC treebank in which the conversation genre (40%) was greater than that of the other written language treebanks (about 25–32%). The results of this study demonstrate that there may be some differences in the dependency direction between the conversational and written language corpora of Chinese. However, whether these differences can be seen as a criterion to judge text genre or not needs further study. Wang and Liu (2017), based on ten dependency treebanks of different genres in the British National Corpus (BNC), selected written- to-be-spoken consisting of scripted television materials and play scripts instead of spoken language, and the data of the study showed that the head-initial (HI) dependency percentages of written-to-be-spoken (51.12%) was greater than that of other treebanks of written language (46.35–49.71%). However, it should be noted that there is a difference between unscripted spoken texts and scripted texts (Wagner, 2014). The written-to-be-spoken selected for this study may not present the true characteristics of spoken language.

From a modality perspective^2^, it is possible to identify the gaps in typological studies that used dependency direction as a measure. In the study of native speaker corpus, Liu (2009b) found differences in the dependency direction between conversational treebanks and written language treebanks, but there is a lack of follow-up studies to further confirm this. In contrast, typological studies of interlanguage have focused on the written modality, and there are gaps in studies specifically on the spoken modality and in comparative studies of the spoken and written modalities. Therefore, does the distribution of the dependency direction of the interlanguage also show differences in different modalities? Moreover, can dependency direction, as an indicator of typological development in the interlanguage written modality, continue to play a role in the spoken modality? These questions deserve further exploration.

In order to fill the gaps of the above studies, this study aims to explore the spoken and written corpora of ECSL learners with the spoken and written corpora of Chinese and English native speakers taken as the reference, and to examine the development patterns of the overall typological features of the interlanguage and the typological features of the important linguistic structures (subjects, objects, attributives, and adverbials) from a cross-modal perspective. At the same time, the typological features of the Chinese and English native speaker corpora under different modalities are examined to further verify the findings in Liu (2009b)'s paper. The specific research questions are as follows:

1. For Chinese and English native speakers, do the typological features of written modality differ from those of the spoken one when measured with the indicator of dependency direction?
2. What is the developmental trend of the typological features in the ECSL learners' interlanguage as L2 proficiency increases?
3. Is the dependency direction applicable to reflecting the typological developmental features across modalities of the ESCL learners' interlanguage?
4. What are the similarities and differences between the typological features of various modalities of the ECSL learners' interlanguage?

## Materials and methods

### Materials

According to the research objectives, we constructed a Chinese interlanguage treebank of different modalities and L2 proficiencies, as well as treebanks of Chinese and English native speakers of different modalities. In the Chinese interlanguage treebank, the spoken language sub-corpus came from the spoken Chinese interlanguage corpus of Nanjing University and the spoken Chinese interlanguage corpus created by Li (2021), both presented in the form of a dialogue between a native Chinese speaker and a second language learner of Chinese. In processing we selected only the corpus of the second language speaker of Chinese, with a total of 39,882 tokens (excluding punctuation). The written language sub-corpus came from the HSK dynamic composition corpus the genre in which is narrative, with a total of 36,618 tokens (excluding punctuation). The topics of the spoken and written materials were all related to the learners' daily life, such as “my daily life,” “the people I know best,” “my hometown,” etc., and the native language of these Chinese learners was English, as shown in Table 1. In the Chinese native language treebank, the spoken corpus came from the CCTV talk show *To Tell the Truth*, with a total of 7,737 tokens (excluding punctuation); the reference data of dependency direction of written language were from Hao et al. (2021). In the English native language treebank, the spoken sub-corpus came from the English talk show with 5,090 tokens; the reference data of dependency direction of writing language were from Jiang et al. (2019).

**Table 1:** A profile of source material.

**Spoken**			**Written**			
**L2 proficiency**	**Number of texts**	**Tokens**	**L2 proficiency**	**Number of texts**	**Tokens**	**Scores**
O1	60	6,581	W1	37	5,306	[50,59]
O2	34	5,725	W2	29	5,469	[60,64]
O3	28	5,493	W3	28	5,341	[65,69]
O4	27	5,706	W4	24	4,900	[70,74]
O5	22	5,320	W5	23	5,150	[75,79]
O6	23	5,502	W6	22	5,170	[80,84]
O7	23	5,555	W7	22	5,282	[85,89]

### Procedure

The corpus was automatically annotated by a computer program and the texts in the corpus were manually pre-processed. Traditional Chinese characters were converted into simplified Chinese characters, and the orthographical mistakes were corrected, but grammatical errors and lexical ambiguities were retained. In the written sub-corpus, some students were not very good at using punctuation marks, and we need to re-break the sentences and assign the correct punctuation marks; in the spoken sub-corpus, many transcribed texts did not have punctuation marks, and again we needed to assign the correct punctuation marks.

The scoring of the corpus was conducted by Chinese teachers with a master's degree in linguistics according to a unified scoring standard. The scoring standards included both the content of expression and the use of language. The former was based on the lexical richness, the effectiveness in communication and the fluency of expression. The latter was based on the grammaticality and structural variety of sentences. Due to the specificity of speaking, the learners' pauses, repetitions and code-switching in utterances were also taken into account in scoring, ensuring that each discourse was scored by at least three people, and the results were compared. The scores of discourses with small differences were averaged. When there was a large difference in the scores of some parts of speech, all the scorers discussed before they reached an agreement on re-scoring. Based on the final scoring results, both the spoken and written corpora were divided into seven grades, ensuring that the number of words in each grade was in the same range, ranging from 5,500 to 6,000 words (excluding punctuation).

After that, we automated the part of speech annotation and dependency relation annotation with Language Technology Platform (LTP) (Che et al., 2010), and then the annotation results were manually checked by referring to the Chinese syntactic annotation system of Liu (2009a).

### Data analysis

**Figure 1:**
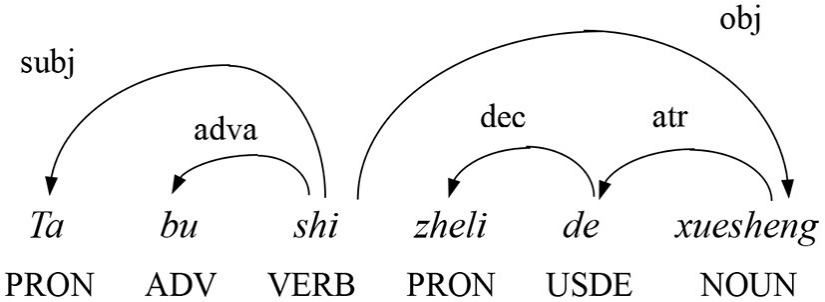
Dependency structure of the sample Chinese sentence *Ta bu shi zheli de xuesheng* (“He's not a student here.”).

The syntactic annotation in this study was conducted within the frame of dependency grammar. The dependency relation, representing an asymmetric binary linear structural relationship between two linguistic units –the governor and the dependent—is fundamental to dependency grammar. Figure 1 shows the dependency relation for the example sentence “He is not a student here,” and the directed arc from the governor to the dependent indicates that the relationship between the two units is asymmetrical and directed. All words in Figure 1 are connected by dependency relations, that is, the subject “he” is subordinate to the tense verb “is.” Figure 1 also reveals that the dependent can either precede or follow the governor in a sentence's linear sequence, which is called the dependency direction of a syntactic dependency relation. If the governor precedes the dependent, then the dependency relation is head-initial (HI); conversely, if the dependent precedes the governor, then the dependency relation is head-final (HF). This can reflect the linear order of linguistic grammatical units. For example, according to the dependency direction of *subj* (subject), the dependency direction between the dependent subject “he” and the governor verb “is” is HF; on the contrary, the dependency direction between the dependent *obj* (object) “student” and the governor verb “is” is HI. By annotating and analyzing all the dependencies, we can calculate the distribution of HF and HI in the sentences or in the whole corpus.

To facilitate the calculation, the annotations in Figure 1 can be converted into Table 2. In Table 2, each row has a set of dependencies, including the dependent word, the governor word, dependency type, the dependency distance and the dependency direction. The difference between two numbers indicating the linear distance between the governor and dependent words is the dependency distance. When the dependent word precedes the governor, the dependency distance is positive and the dependency direction is HF; otherwise the dependency direction is HI. Therefore, the dependency direction can be determined by the positive or negative value of the dependency distance. The dependency direction distribution is calculated according to the formula given by (Liu, 2010), which can help us to calculate the percentage distribution of dependency direction (HF or HI) for a specific dependency relation or the whole corpus. The formula (Liu, 2010) is as follows:


(1) $$\begin{array}{l} \text{Percentage of head}-\text{final dependency}\\ \;=\frac{\text{frequencies of the head }-\text{ final dependencies}}{\text{total number of dependencies in the treebank}}\times \;100 \end{array}$$

(2) $$\begin{array}{l} \text{Percentage of head}-\text{initial dependency}\\ \;=\;\frac{\text{frequencies of the head }-\text{ initial dependencies}}{\text{total number of dependencies in the treebank}}\times \;100 \end{array}$$

Using the above formula, it is possible to calculate the percentage distribution of HF and HI in the overall or the important linguistic structures of Chinese learners from native English backgrounds in different modalities, and thus to study the development of their typological characteristics.

**Table 2:** Annotation of the sample sentence in Chinese, *Ta bu shi zheli de xuesheng* (“He's not a student here.”).

**Order**	**Dependent**	**POS**	**Order**	**Governor**	**POS**	**Dependency**	**Dependency**	**Dependency**
**number**	**word**		**number**	**word**		**type**	**distance**	**direction**
1	*他 (Ta)*/He	r	3	*是 (shi)*/is	vl	subj	2	HF
2	*不 (bu)*/not	d	3	*是 (shi)*/is	vl	adva	1	HF
3	*是 (shi)*/is	vl	7	。	bjd	s	0	/
4	*这里 (zheli)*/here	r	5	*的 (de)*/of	usde	dec	1	HF
5	*的 (de)*/of	usde	6	*学生 (xuesheng)* / student	n	atr	1	HF
6	*学生 (xuesheng)*/student	n	3	*是 (shi)*/is	vl	obj	−3	HI
7	。							

## Results and discussion

### The overall typological features of Chinese interlanguage

**Figure 2:**
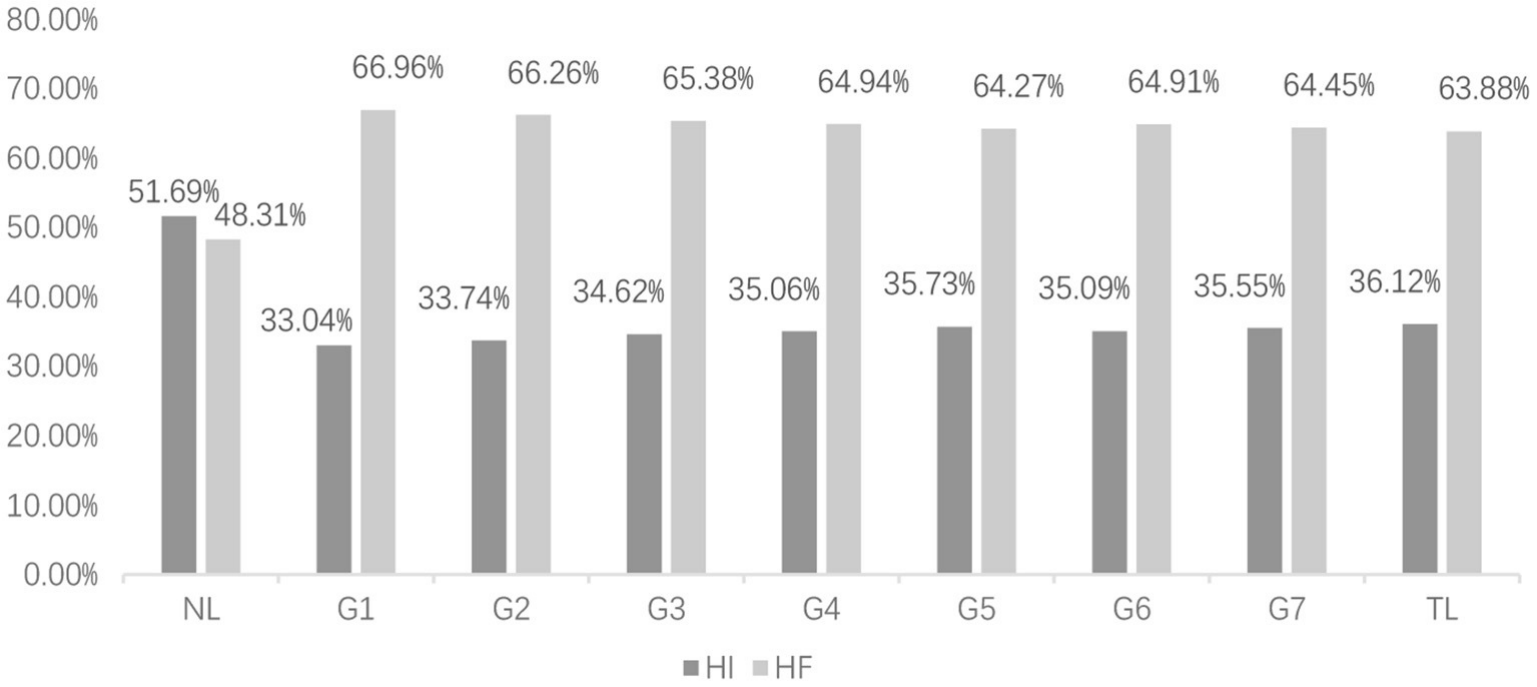
In written modality, distribution of ECSL learners' dependency directions at each level and in the contrastive treebanks. NL, native language; TL, target language.

**Figure 3:**
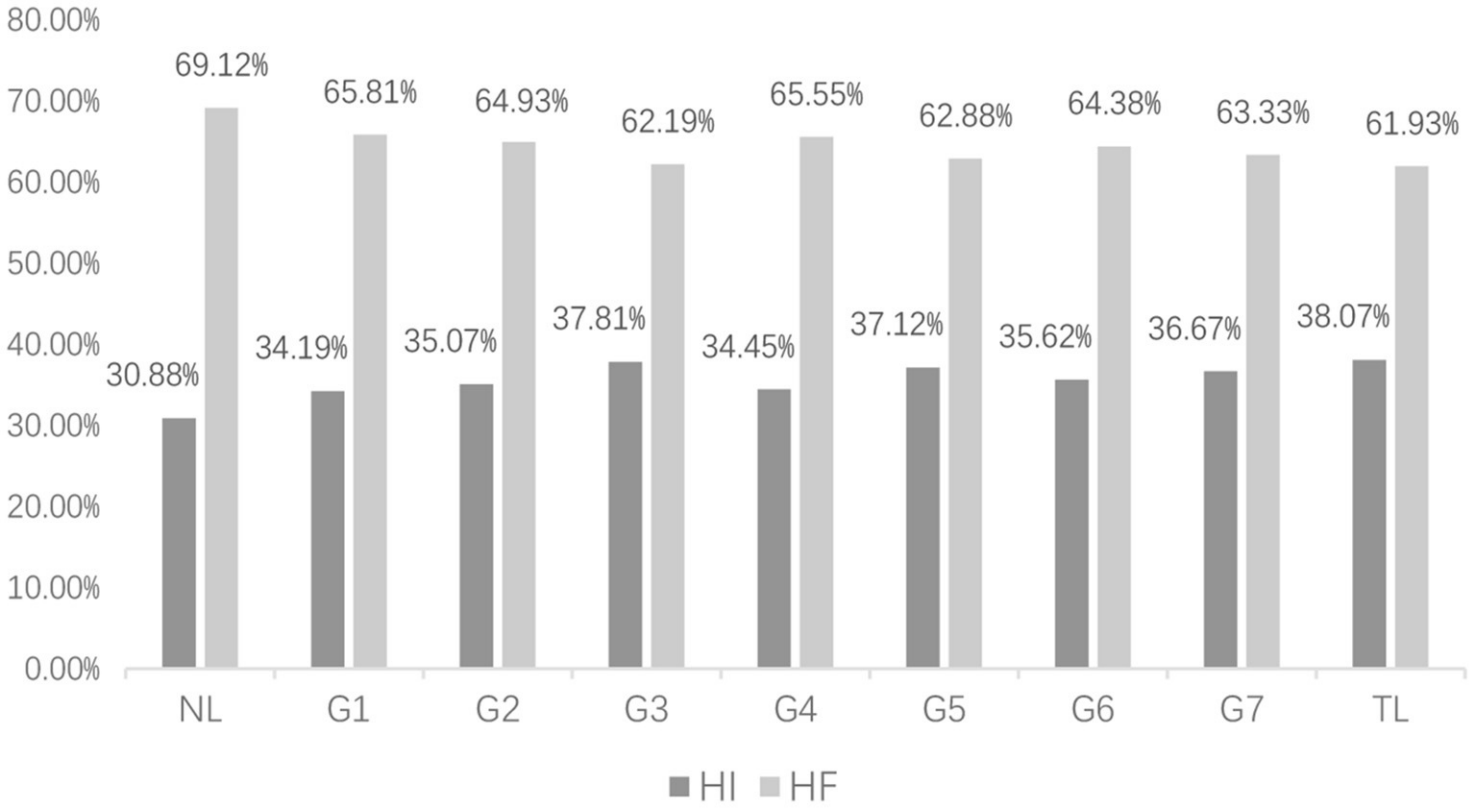
In spoken modality, distribution of ECSL learners' dependency directions at each level and in the contrastive treebanks.

Figure 2 shows the distribution of the seven grades of ECSL learners' interlanguage and the dependency direction of English (NL) and Chinese (TL) in the written modality; Figure 3 shows the distribution of the seven grades of ECSL learners' interlanguage and the dependency direction of English and Chinese in the spoken modality.

As shown in Figures 2, 3, the HI dependency percentage (HI%) for Chinese native speakers was 36.12% in the written modality and 38.07% in the spoken modality. This result is consistent with Liu (2009b)'s finding that the HF dependency percentage (HF%) in the spoken modality is greater than that in the written modality in the Chinese native speaker corpus. The HI dependency percentage for native English speakers was 51.69% in the written modality and 30.88% in the spoken modality. However, the HI% in the spoken modality is much smaller than that in the written modality, which is inconsistent with the findings of Wang and Liu (2017). This is possibly due to the fact that the source material is written-to-be-spoken instead of spoken language. Written-to-be-spoken, that is scripted down and then presented orally, is colloquial, but it undergoes the process of writing, revising, and embellishing, which differs from unplanned spoken language. And as we mentioned above, the characteristics of unplanned spoken language are closer to those of typical spoken language (Ochs, 1979). Moreover, it can be observed that native English speakers differ more in the HF dependency percentage (HF%) between written and spoken language than native Chinese speakers. By examining the native speaker data, we can answer Question 1: it can be concluded that both native Chinese and native English differ in their typological characteristics across modalities, and compared with Chinese native speakers, English native speakers show more obvious differences in different modalities.

Does the finding that the typological characteristics of Chinese and English native languages show differences across modalities apply to Chinese interlanguage as well? Looking at the dependency direction of Chinese interlanguage in different modalities, we found that the HI dependency percentages in spoken modality at all other levels were larger than those in the written modality, except for spoken modality in G4 (34.45%), which was smaller than that in the written modality (35.06%). Therefore, the typological characteristics of Chinese interlanguage in different modalities show differences, and the differences are closer to the target language (Chinese), that is, the HI dependency percentage of spoken modality is greater than that of written modality.

To answer Questions 2 and 3, further comparison of the developmental trends in the typological characteristics of interlanguage in different modalities follows. As shown in Figure 2, the HI% of ECSL learners in the written modality increased from 33.04% in G1 to 35.55% in G7 as L2 proficiency increased, gradually approaching 36.12% in Chinese (TL). The statistical analysis shows that the HI dependency percentage of ECSL learners was proportional to language proficiency in the written modality; the regression equation is *y* = 0.4*x* + 33.1, *p* = 0.007, *R*^2^ = 0.759. The results indicate that the dependency direction in the written modality was related to learners' language proficiency. This finding is consistent with the previous studies on written modality (Jiang et al., 2019; Hao et al., 2021), which means that the dependency direction can be used as a reliable indicator of learners' language proficiency in the written modality. However, it is worth noting that the HI% of ECSL learners from G1 to G3 were 33.04%, 33.74%, and 34.62%, which were closer to the target language Chinese (36.12%) compared to the learners' native English (51.69%). The typological characteristics of the ECSL learners with lower Chinese proficiency can be well approximated to the target language typological characteristics and seem to be less influenced by negative native language transfer, which is not the same as the findings reported by previous studies on the written modality. This is because in addition to native language transfer, many other factors such as age, language learning environment and conceptual transfer may also influence the formative period of language acquisition (Diane, 1997; Jarvis, 2016). The development of an interlanguage is a complex process, and interlanguage cannot be viewed singularly as a hybrid language formed under the influence of the native language (Saville-Troike, 2008). In addition, it was found that the HF% at the G1 was the highest among the seven grades, even higher than the TL. With the improvement of language proficiency, HF% gradually decreased and finally approached TL. We speculate that ECSL learners with less proficiency in the language may display an “overcorrection” tendency. As the language level increases, this effect gradually decreases and converges to the target language level.

As shown in Figure 3, the HF% of ECSL learners in the spoken modality fluctuated more and did not show a significant pattern as the language proficiency increased, but the dependency direction for the seven grades was always between the ECSL learners' native language and the target language. The regression equation is *y* = 0.28*x* + 34.726, *p* = 0.326, *R*^2^ = 0.030. This indicates that there was no statistically significant correlation between the dependency direction and ECSL learners' language proficiency in the spoken modality. It is noteworthy that in the spoken modality the HF% of Chinese learners (62.19–65.81%) exceeded that of the target language (61.93%) in all language grades, and the HI% (34.19–37.81%) was lower than that of the target language Chinese (38.07%). Relatively speaking, this tendency of ECSL learners' having a higher HF% and lower HI% was closer to their native language of English (HF%: 69.12%; HI%: 30.88%). The typological features of the spoken modality corpus seem to be more susceptible to negative native language transfer and may show more typological errors than the written modality.

Here it is possible to answer Questions 2 and 3 that the developmental typological trend of Chinese interlanguage differs across modalities, and that the dependency direction is not appropriate for measuring the development of interlanguage in the spoken modality; it does not seem to work as a generic indicator of typology across modalities. There may be two reasons why the dependency direction, a reliable indicator that can measure changes in the typological features of interlanguage in the written modality, does not work in the spoken modality.

First of all, a very important distinction between the two modalities is the difference in the time pressure to which learners are subject. Successful writers need to effectively manage and well coordinate the planning, transcription and revision subprocesses that compete for the limited cognitive resources (e.g., working memory) needed in writing (Mccutchen et al., 1994; Kellogg, 2001), and this competition may be exacerbated under testing conditions with time pressure (Worden, 2009; Gong et al., 2022). Although writing time is also limited in the test setting, writers can determine their own writing speeds. In order to achieve discourse coherence, speakers need to keep uttering words. Therefore, the speakers are actually faced with greater temporal pressure related to the cognitive load compared to the learners' performance of the written language. Due to greater time pressure, speakers' attention is allocated between conceptualizing information and linguistic encoding, which requires online processing, that is, planning their content while outputting content and linguistic forms (Yuan and Ellis, 2003). However, the time spent on planning information is also integrated into the writing process; writers can use more time for information retrieval and planning and also have more attentional resources available to revise their output while coding (Ochs, 1979; Grabe and Kaplan, 1996; Grabowski, 2007). Additionally, Grabowski (2007) has stated that L2 writers can better control the use of language and have better access to knowledge stored in long-term memory compared to speakers. Learners can use both explicit and tacit knowledge in writing, and it is tacit knowledge that is most often used in spoken language. On this basis, Grabowski (2007) has argued that second language learners can show a more complete store of second language knowledge in the written modality than in the spoken modality, with higher accuracy and lexical richness often being manifested in writing, which has also been confirmed by subsequent studies (Kormos and Trebits, 2012; Zalbidea, 2017; Cho, 2018).

Secondly, compared to written language, which emphasizes grammatical norms, speakers are in immediate face-to-face communication scenarios, leading them to speak with more emphasis on the expression of meaning and less attention to linguistic forms, such as irregular use of word order. We found that a typical manifestation of this is the inversion of components in spoken Chinese, that is, often the key message is expressed, and then some necessary content is supplemented later. This is because the speaker has limited time to think and will choose the important part to say first to get the listener's attention. For example, a speaker might say *Chifan le wo* (“I've had a meal.”), which is a subject-postposition inversion, mainly to emphasize the action of *chifan* (“to eat something”). The dependency direction reflects the word order, and inverted sentences can occur in all grades of learners' speech, and the reversal of order caused by them will affect the dependency direction in the spoken modality.

Thus, due to the difference in output processes and time constraints, the spoken output is more difficult, less accurate than the written modality in terms of language accuracy, and may have more typological errors. Due to the greater time pressure and cognitive load on spoken language, learners in the spoken modality appear to be more susceptible to negative transfer from their native language than in the written modality. Finally, possible component inversions in the spoken modality could likewise affect changes in the dependency direction. We speculate that the above may be one of the reasons why dependency direction is a valid developmental typology indicator but loses its role in the spoken modality.

### The typological developmental features of important linguistic structures in interlanguage

In this sub-section, in order to answer Question 4 and further verify the above speculation, the following part examines the typological development characteristics of four important syntactic structures: subjects, objects, attributives, and adverbials. These four syntactic structures were selected because they are most frequently used in both Chinese and English and are important linguistic structures that constitute major syntactic relations. Although both Chinese and English are SVO languages and their basic word orders are very similar, there are differences in the placement of attributives and adverbials. Therefore, in the next sub-sections we will analyze and discuss the distribution of the four linguistic structures of the written and spoken modalities in terms of their dependency directions.

#### The typological developmental features of subjects and objects

**Figure 4:**
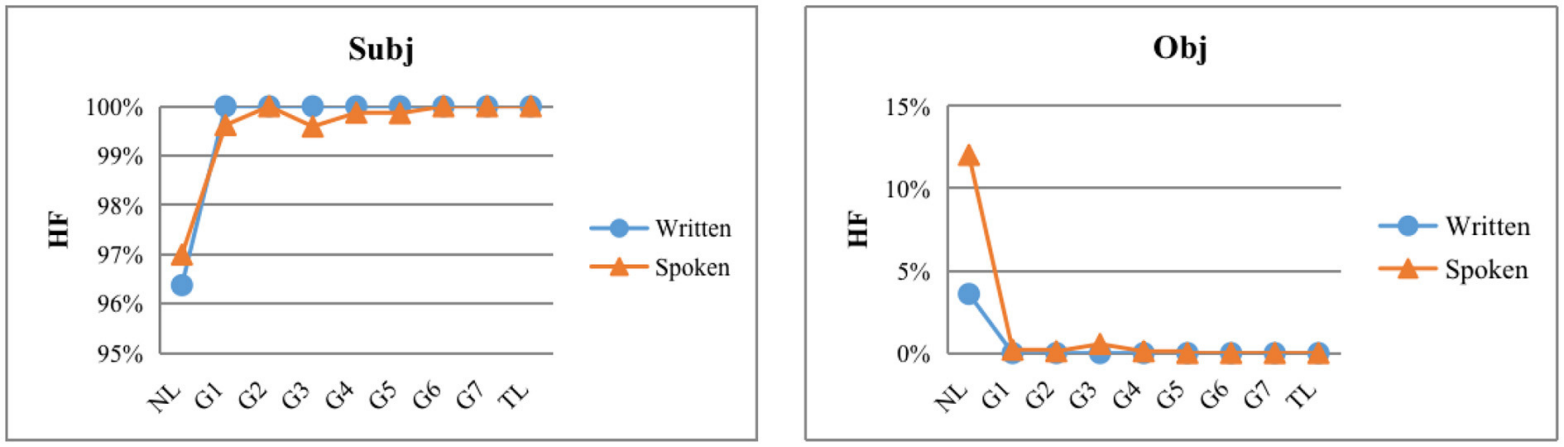
HF dependency direction distributions of subjects and objects at each level and in contrastive treebanks.

As shown in Figure 4, the HF% of subject language for ECSL learners of all levels of language proficiency in the written modality was 100%, and the HF% of object language was 0%, which was consistent with the native Chinese speakers. In the spoken modality, although the subject dependency direction of the elementary level Chinese learners differed from that of the native Chinese speakers, they all showed the same HF tendency as the TL, with HF% exceeding 99% and approaching 100% (HF% of the subject in the TL); the object dependency direction of the elementary level Chinese learners also differed from that of the native Chinese speakers, but they all showed the same HI. The object dependency direction also differed from that of Chinese native speakers, but they all show the same HI tendency as TL, with HI% exceeding 99% and approaching 100% (object: HI% in TL).

As shown in Table 3, in the written modality, the HF% of subject and HI% of object did not change as the language level increased, and always remained the same as native Chinese speakers. Apparently, there was no correlation between the change in HF% of subject and HI% of object and language level in the written modality. As shown in Table 4, in the spoken modality, as the language level increased, the HF% of ECSL learners' subject language increased from 99.62% in G1 to 100% in G2, then decreased to 99.59% in G3, then increased to 99.86% in G5, and finally reached 100% in G6. Linear regression results showed that there was no significant correlation between the HF% of subject language and language level in the spoken modality (*p* = 0.143). The HI% of ECSL learners' object changed from 99.80% in G1 to 99.89% in G4 and finally reached 100% in G5, and the linear regression results showed that the HI% of the spoken modality's object was not significantly correlated with language proficiency (*p* = 0.147).

**Table 3:** In written modality, frequencies and dependency direction distributions of subjects and objects at each level and in contrastive treebanks.

	**subj**			**obj**		
	**Frequency**	**HF**	**HI**	**Frequency**	**HF**	**HI**
NL	718 (13.35%)	692 (96.38%)	26 (3.62%)	390 (7.25%)	14 (3.59%)	376 (96.41%)
G1	614 (11.57%)	614 (100%)	0 (0.00%)	830 (15.64%)	0 (0.00%)	830 (100%)
G2	626 (11.45%)	626 (100%)	0 (0.00%)	915 (16.73%)	0 (0.00%)	915 (100%)
G3	587 (10.99%)	587 (100%)	0 (0.00%)	851 (15.93%)	0 (0.00%)	851 (100%)
G4	525 (10.71%)	525 (100%)	0 (0.00%)	817 (16.67%)	0 (0.00%)	817 (100%)
G5	513 (9.96%)	513 (100%)	0 (0.00%)	845 (16.41%)	0 (0.00%)	845 (100%)
G6	540 (10.44%)	540 (100%)	0 (0.00%)	770 (14.89%)	0 (0.00%)	770 (100%)
G7	493 (9.33%)	493 (100%)	0 (0.00%)	872 (16.51%)	0 (0.00%)	872 (100%)
TL	977 (9.55%)	977 (100%)	0 (0.00%)	1,903 (18.60%)	0 (0.00%)	1,903 (100.%)

**Table 4:** In spoken modality, frequencies and dependency direction distributions of subjects and objects at each level and in contrastive treebanks.

	**subj**			**obj**		
	**Frequency**	**HF**	**HI**	**Frequency**	**HF**	**HI**
NL	666 (13.08%)	646 (97.00%)	20 (3.00%)	632 (12.42%)	76 (12.03%)	556 (87.97%)
G1	1,050 (15.96%)	1,046 (99.62%)	4 (0.38%)	981 (14.91%)	2 (0.20%)	978 (99.80%)
G2	845 (14.76%)	845 (100%)	0 (0.00%)	910 (15.90%)	1 (0.11%)	909 (99.89%)
G3	740 (13.47%)	737 (99.59%)	3 (0.41%)	911 (16.58%)	5 (0.55%)	906 (99.45%)
G4	781 (13.69%)	780 (99.87%)	1 (0.13%)	900 (15.77%)	1 (0.11%)	899 (99.89%)
G5	739 (13.89%)	738 (99.86%)	1 (0.14%)	862 (16.20%)	0 (0.00%)	862 (100%)
G6	692 (12.58%)	692 (100%)	0 (0.00%)	873 (15.87%)	0 (0.00%)	873 (100%)
G7	592 (10.66%)	592 (100%)	0 (0.00%)	788 (14.19%)	0 (0.00%)	788 (100%)
TL	805 (11.09%)	805 (100%)	0 (0.00%)	1,011 (13.93%)	0 (0.00%)	1,011 (100%)

The more similar the target language is to the language type of the learner's native language, the better the acquisition by the learner, a claim that has been confirmed by previous studies (Jansen et al., 1981; Mangana, 2002; Jach, 2018). Both Chinese (TL) and English (NL) are SVO languages, and both have similar subject-verb (SV) and verb-object (VO) structures, which are easier for learners to acquire. Therefore, according to our findings, Chinese learners in the penultimate modality showed typological features consistent with native Chinese speakers at all language levels, both in terms of the distribution of dependency directions of subject-predicate and verb-object structures; ECSL learners in the spoken modality, although they differed from those of the native Chinese speakers in terms of typological features at the elementary level, the differences were smaller and they eventually reached the level of Chinese (TL).

The data here can confirm the previous speculation that although the typological features of subject-verb and verb-object structures are relatively easy to acquire, the greater temporal pressure and cognitive load in the spoken modality may make ECSL learners more prone to word order errors than in the written modality. In the spoken modality, ECSL learners' subject-verb structure errors are shown in Example (1–2). In (1), the ECSL learner put the subject *huǒchē* (“train”) after the verb *sh*ì (“is”); in (2), the ECSL learner placed the subject *zuògōng* (“made”) after the predicate “very well,” which does not conform to the canonical order of the Chinese subject-verb structure. It was found that in (1), the subject-verb found acts as the object, and in (2), the subject-verb structure acts as the predicate, and this complex syntax may have caused difficulties for ECSL learners, thus leading to errors. In the spoken modality, the ECSL learners' verb-object structure errors are shown in (3–5). In (3), the ECSL learner placed the object *zhuānyè* (“major”) before the verb *xuefor* (“*choose*”); in (4), the ECSL learner placed the object *wènt*í (“questions”) before the verb *hu*í*dá* (“answer”); in (5), the ECSL learners put the object *yuègu(5)* (“moonlight”) before *méiyor* (“no”), which does not conform to the canonical order of the Chinese verb-object structure. Looking at all the ECSL learners' errors in subject-verb and verb-object structures, we found that: in subject-verb structures, ECSL learners made fewer word order errors, and if they did, the errors occurred more often in sentences with complex structures; in verb-object structures, ECSL learners made more order errors, even in simple sentences.

(1) ^*^Wǒ juéde kěnéng **shì** huǒchē sāngè xiǎoshí. (O3, t203a, s20^3^)
  I think maybe **is** train three hour
  “I think maybe it takes three hours by train.”
  “Wǒ juéde kěnéng huǒchē **shì** sāngè xiǎoshí.”

(2) ^*^Zhège yifu **hěnhǎo** zuògōng. (O4, t62b, s2)
  This dress **perfectly** made
  “This dress is **perfectly** made.”
  “Zhège yı̄fu zuògōng **hěnhǎo**.”

(3) ^*^Wode zhuānyè wǒ **xuǎnzé**. (O1, t16, s3)
  My major I **choose**
  “I **choose** my (own) major.”
  “Wǒ **xuǎnzé** wǒde zhuānyè.”

(4) ^*^Xiànzài wo sānge wèntí **hu**í**dá**.(O2, t131, s1)
  Now I three questions **answer**
  “Now I'll **answer** three questions.”
  “Xiànzài wo **hu**í**dá** sānge wèntí.”

(5) ^*^Nánjı̄ng yuèguāng **méiyǒu**. (O3, t123, s5)
  Nánjı̄ng moonlight **no**
  “There is **no** moonlight in Nanjing.”
  “Nánjı̄ng **méiyǒu** yuèguāng.”

In addition, we were able to verify the previous hypothesis that the inversion phenomenon, which is unique to the spoken modality compared to the written modality, also affects the role of the indicator of dependency direction as a measure of linguistic typological features. In the subject-verb structure, ECSL learners inverted the subject as shown in (6–8), placing the subjects *Dékèsàsj* (“Texas”), *che zhèzhs”j dhèzhs* (“eat this kind of food”) and *nàxie làxiet àpùzht* (“those foreigners uploaders”) after the predicate; in the verb-object structure, ECSL learners inverted the verb as shown in (9), placing the verb *kafter* (“possible”) after the object.

(6) Hěn rè Dékèsàsi^4^. (O1, t25, s3)
  Very hot Texas
  “It's very hot in Texas.”

(7) Jiù hěn nánshòu chi zhèzhǒng dōngxi. (O3, t200a18, s1)
  Just very hard eat this kind of food
  “It's just hard to eat this kind of food.”

(8) Tāmen shuō yǒu hěnduō fěnsı̄ nàxie lǎowài àpùzhǔ. (O5, t209b4, s1)
  They say have a lot of fan those foreigner uploaders
  “They said that those foreigner uploaders have a lot of fans.”

(9) Yùndòng bù kěnéng. (O1, t91, s8)
  Exercise no possible
  “It is impossible to exercise.”

#### The typological developmental features of attributives

**Figure 5:**
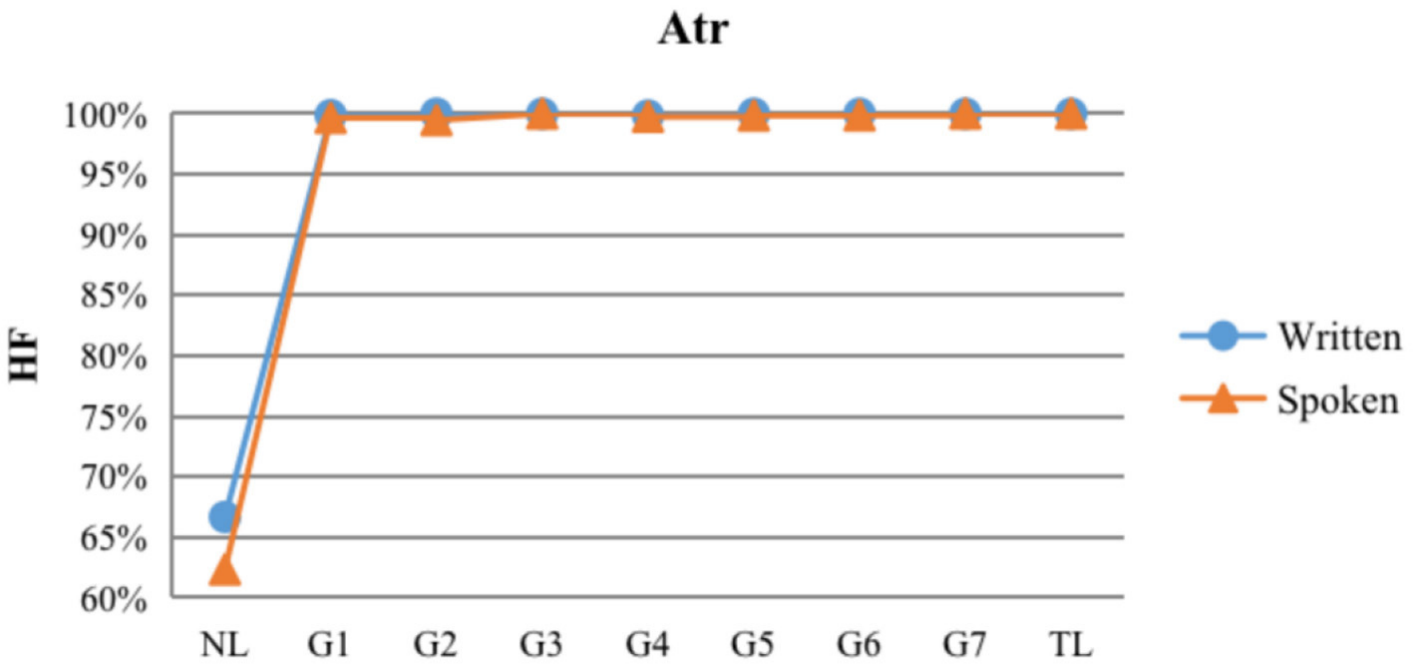
HF dependency direction distributions of attributives at each level and in contrastive treebanks.

As shown in Figure 5, Table 5, in written modality, the dependency direction showed a high HF% tendency at the beginning, reaching 99.88% in G1, and reached 100% in all subsequent stages except for G4, where the HF% was 99.85%, consistent with TL (Chinese). In spoken modality, the dependency direction also showed a higher tendency of HF%, varying from 99.65% in G1 to 99.70% in G6, with HF% reaching 100% in G3, and later 100% once again in the final G7 stage. Linear regression results showed that the HF% of attributives did not correlate with language level in either the written or spoken modality (*ps* > 0.05).

**Table 5:** Frequencies frequencies and dependency direction distributions of attributives at each level and in contrastive treebanks.

	**Written**			**Spoken**		
	**Frequency**	**HF**	**HI**	**Frequency**	**HF**	**HI**
NL	801 (14.89%)	534 (66.67%)	267 (33.33%)	600 (11.79%)	374 (62.33%)	226 (37.67%)
G1	855 (16.11%)	854 (99.88%)	1 (0.12%)	850 (12.92%)	847 (99.65%)	3 (0.35%)
G2	828 (15.14%)	828 (100%)	0 (0.00%)	739 (12.91%)	735 (99.46%)	4 (0.54%)
G3	808 (15.13%)	808 (100%)	0 (0.00%)	680 (12.38%)	680 (100%)	0 (0.00%)
G4	646 (13.18%)	645 (99.85%)	1 (0.15%)	770 (13.49%)	768 (99.74%)	2 (0.26%)
G5	727 (14.12%)	727 (100%)	0 (0.00%)	619 (11.64%)	618 (99.84%)	1 (0.16%)
G6	678 (13.11%)	678 (100%)	0 (0.00%)	675 (12.27%)	674 (99.85%)	1 (0.15%)
G7	791 (14.98%)	791 (100%)	0 (0.00%)	678 (12.21%)	678 (100%)	0 (0.00%)
TL	2,213 (21.63%)	2,213 (100%)	0 (0.00%)	970 (13.37%)	970 (100%)	0 (0.00%)

Unlike the subject-verb and verb-object structures, the position of the attributive structure differs greatly between Chinese and English. The attributive structure in Chinese is located before the modified noun (Written HF:100%; Spoken HF:100%), while the position of the attributive structure in English is flexible and can be before or after the modified noun (Written HF: 66.67%; Spoken HF: 62.33%). As noted earlier, differences in language type may affect acquisition, so learners may have more difficulty mastering the attributive structure than the subject-verb and verb-object structures. From Table 5, it can be seen that in the written modality, unlike the dependency direction of the subject and object which always remained consistent with the TL, ECSL learners made inflectional errors in stages G1 and G4; in the spoken modality, the dependency direction of the subject reached the TL level in stage G6 and the dependency direction of the object reached the TL level in stage G5, while the ECSL learners' definite HF%, although reaching 100% in stage G3, still fluctuated afterwards until the final stage G7.

Although ECSL learners were more likely to make errors in the word order of the attributive structure compared to the first two syntactic structures, the error rate was not high. In the written modality, ECSL learners' attributive HF% reached 99.88% at the G1 stage, and 100% at all stages except for the G4 stage, where HF% was 99.85%, in the spoken modality, ECSL learners' HF% reached 99.65% at the G1 stage, and the HF% at the final stage was consistent with TL. The reason for this situation may be related to the single position of the attributive in Chinese, where the dependent word used as the attributive is always located before the governor (HF%: 100%). According to Eckman (1977)'s tokenization hypothesis, second language learners have more difficulty in learning the more tokenized syntactic structures. ECSL Learners only need to combine two governor word positions in NL into one position, which is considered to be an unmarked and less difficult language item. The usage-based language learning theory suggests that second language learning is driven by the learner's experience with language use and input (Ellis et al., 2016). ECSL learners may be influenced by the 100% backwardness of the governor of the attributive structure in TL when adjusting the word order of the Chinese attributive structure, and usually draw less on the grammatical rules of NL. Therefore, the attributive is not a difficult structure for ECSL learners of both modalities to master.

Although the Chinese attributive structures are unmarked and less difficult language items, ECSL learners are more likely to make linguistic typological errors in the attributive structures than in the subject-verb and verb-object structures, which are consistent with the English-Chinese order. For example, in the subject-verb and verb-object structures, ECSL learners in the written modality did not make errors in word order, while in the attributive structure they were more likely to be affected by negative native language transfer and made a small number of word order errors. As shown in (10–11), ECSL learners were influenced by the typological features of the attributive structure in their NL (English) and placed the attributive after the modified noun, which does not conform to the word order of the TL (Chinese) attributive structure. Similarly, in the spoken modality, ECSL learners were more likely to make word order errors in the attributive structure due to the negative transfer caused by the more flexible position of the attributive in the NL (English), and there were more word order errors in the fixation structure in the spoken modality compared to the written modality, as shown in (12–13). It is also worth noting that not all of the word order errors of the attributive structure in the spoken modality can be explained by negative native language transfer, as shown in (14). In the NL (English), *tèbié chuántlex* (“special tradition”) is also a structure in which the attributive word precedes the modified noun, but ECSL learners still placed the attributive word *tèbié* (“special”) after the modified noun *chuánt th* (“tradition”). This may be related to the time pressure and cognitive load that L2 learners face when speaking, which leads to increased difficulty in speaking. This could verify the previous speculation that ECSL learners in the spoken modality are indeed more prone to word order errors and more likely to show negative native language transfer, which is closely related to the greater time pressure and cognitive load in the spoken modality.

(10) ^*^Yīnwèi tā **yīqiè** zuòde dōu shì fùmǔ suǒ jiāo de. (W1, t519, s9)
  Because he **everything** do all is parents (AUX) teach (AUX)
  “Because **anything** he does is taught by his parents.”
  “Yīnwèi tā zuòde **yīqiè** dōu shì fùmǔ suǒ jiāo de.”

(11) ^*^Wǒ shì yíwèi èrshisuì de **huáyì** Jiānádà. (W4, t566, s1)
  I am a twenty years old (AUX) **Chinese** Canada
  “I am a twenty years old **Chinese** Canadian.”
  “Wǒ shì yíwèi èrshisuì de Jiānádà **huáyì**.”

(12) ^*^Wǒde jiāxiāng zài **Ból**í**n** Déguó^5^. (O1, t82, s1)
  My hometown in **Berlin** Germany
  “My hometown is in **Berlin**, Germany.”
  “Wǒde jiāxiāng zài Déguó **Ból**í**n**.”

(13) ^*^Wǒ rènshi tā zài **Mànchèsītèdàxué** yīngguó. (O5, t149, s2)
  I know her at **University of Manchester** England
  “I know her at the **University of Manchester** in England.”
  “Wǒ rènshi tā zài yīngguó **Mànchèsītèdàxué**.”

(14) ^*^Zài Wǎngshīyuán you tāmen de **chuántǒng** tèbié. (O4, t156, s6)
  In Master-of-Nets Garden have they of **tradition** special
  “They have a special **tradition** in Master-of-Nets Garden.”
  “Zài Wǎngshīyuán you tāmen de tèbié **chuántōng**.”

#### The typological developmental features of adverbials

**Figure 6:**
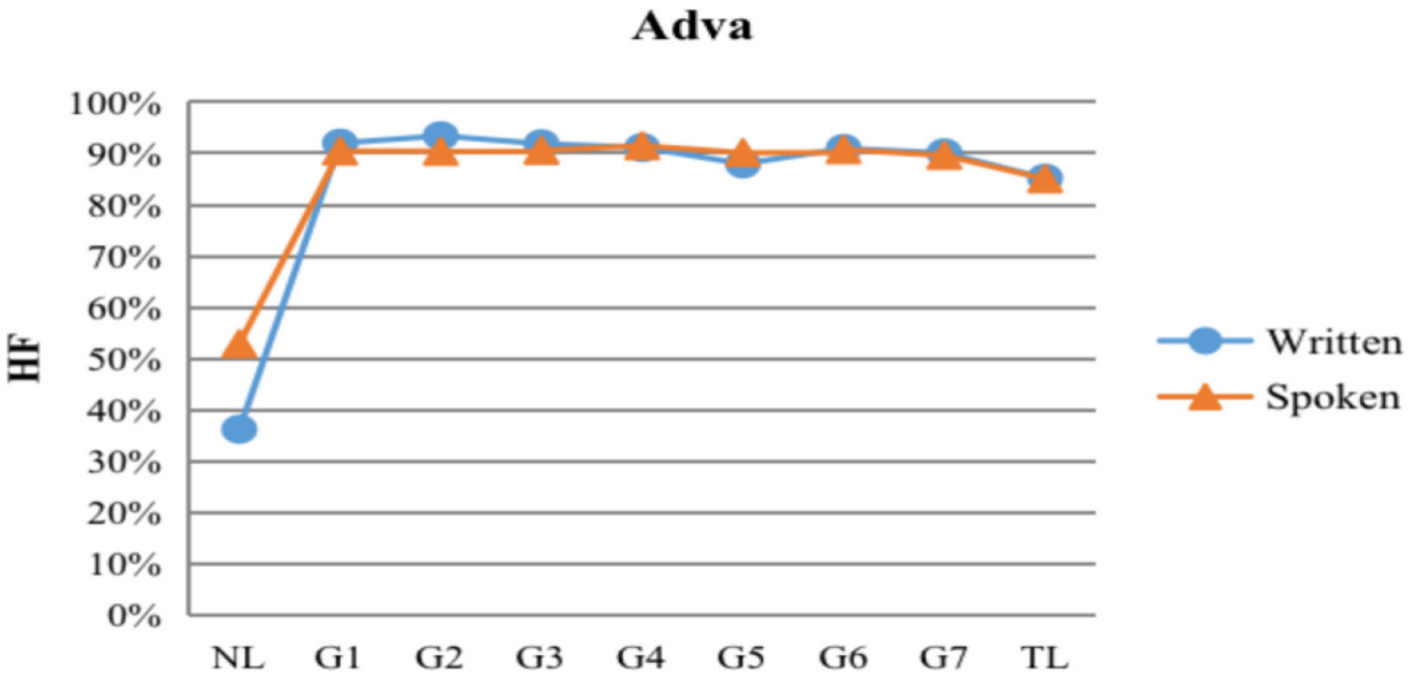
HF dependency direction distributions of adverbials at each grade and in contrastive treebanks.

Traditionally, scholars of Chinese grammar in China have distinguished adverbials and complementations, for example, an adverbial usually precedes a predicate verb, while a complementation follows a predicate verb. However, in descriptive grammars of many other languages, there is no such a distinction which is merely based on the relative position. Therefore, in order to keep consistent with other language studies across the world, all the complementations were classified as adverbials in the current study. Figure 6 shows the dependency direction of adverbials produced by ECSL learners in both modalities. Compared with the three syntactic structures mentioned above, the developmental trend of the adverbial structure is more complex as the language proficiency increases. As shown in Table 6, at the G1 level, the HF% of adverbial in the written and spoken modalities were 91.99 and 90.45%, respectively, which were closer to the HF% in TL Chinese (written: 85.18%; spoken: 85.04%) than in NL English (written: 36.23%; spoken: 52.89%). Moreover, in both modalities, the HF% of adverbials showed a decreasing trend, gradually approaching TL. Linear regression results showed that the HF% of adverbials did not correlate with language proficiency in the written and spoken modalities (*ps*>0.05).

**Table 6:** Frequencies and dependency direction distributions of adverbials at each level and in contrastive treebanks.

	**Written**			**Spoken**		
	**Frequency**	**HF**	**HI**	**Frequency**	**HF**	**HI**
NL	770 (14.32%)	279 (36.23%)	491 (63.77%)	760 (14.93%)	402 (52.89%)	358 (47.11%)
G1	1,024 (19.30%)	942 (91.99%)	82 (8.01%)	1,413 (21.47%)	1,278 (90.45%)	135 (9.55%)
G2	1,035 (18.92%)	967 (93.43%)	68 (6.57%)	1,040 (18.17%)	940 (90.38%)	100 (9.62%)
G3	1,045 (19.57%)	960 (91.87%)	85 (8.13%)	1,030 (18.75%)	933 (90.58%)	97 (9.42%)
G4	1,006 (20.53%)	917 (91.15.%)	89 (8.85%)	1,091 (19.12%)	998 (91.48%)	93 (8.52%)
G5	1,013 (19.67%)	891 (87.96%)	122 (12.04%)	1,014 (19.06%)	914 (90.14%)	100 (9.86%)
G6	1,139 (22.03%)	1,036 (90.96%)	103 (9.04%)	1,068 (19.41%)	969 (90.73%)	99 (9.27%)
G7	1,030 (19.50%)	928 (90.10%)	102 (9.90%)	1,279 (23.02%)	1,146 (89.60%)	133 (10.40%)
TL	1,916 (9.36%)	1,632 (85.18%)	284 (14.82%)	1,604 (22.10%)	1,364 (85.04%)	240 (14.96%)

Compared with the three linguistic structures mentioned above, the HF% of written modality learners was 4.92% higher than TL, and the HF% of spoken modality learners was 4.56% higher than TL at the final G7 stage; there were significant differences in the adverbial typological characteristics in the form between ECSL learners and native Chinese speakers. At the same time, we were able to find that ECSL learners of both modalities showed a higher tendency of HF% at the beginning, even higher than TL (Chinese). The reason for this situation may be that the position of the adverbial is more flexible in both Chinese and English, with the Chinese adverbial tending to precede the governor word (written HF%: 85.18%; spoken HF%: 85.04%), while the English adverbial tends to follow the governor or be on either side of it (written HF%: 36.23%; spoken HF%: 52.89%). There is a complex bidirectional relationship between the typological characteristics of adverbials in both languages, so ECSL learners may struggle with marking difficulties. Therefore, ECSL learners may be influenced by TL high-frequent HF% and tend to rely on this rule when it comes to uncertain expressions. This could explain the facts that ECSL learners presented higher HF% at the elementary level and that HF% gradually decreased as the language proficiency increased, eventually approaching TL.

Although both modal outputs of ECSL learners showed a higher tendency of HF% in adverbial structures, ECSL learners were still inevitably negatively transferred from their native English due to the complex distribution of adverbial positions in their NL (English), which led to a higher number of word order errors. For example, in English, adverbial of time is usually placed at the end of a sentence; but in Chinese, it is placed at the beginning of a sentence or before the verb, as shown in (15), adverbial of time 1949 *nián* (“the year of 1949”) should be placed before the verb *chusheng* (“born”). Another example is that when a prepositional structure is used as adverbial of place in a sentence, it is located at the end of the sentence in English, but before the verb in Chinese, and ECSL learners may be influenced by their native language, leading to inaccurate order, as shown in (16–17). In addition, the adverbial of manner, adverbial of cause, adverbial of degree and accompanying adverbial are usually placed at the end of the sentences in English, while the opposite is true in Chinese, which is also a type of frequently occurring errors for ECSL learners, as shown in (18–22). All the errors in word order revealed that ECSL learners were indeed affected by negative transfer of their native language in the adverbial structures, even more so than in the first three linguistic structures. L2 learners are more likely to have difficulties in acquiring those typological characteristics of the target language that involve more than just syntactic knowledge. From a cognitive perspective, second language acquisition is the process of constructing new conceptual structures and ways of thinking (Robinson and Ellis, 2008). The typological features of gerunds are influenced by pragmatic and cognitive factors, such as information structure, semantic category, and context (Qnirk et al., 1985; Austin et al., 2004; Hasselgård, 2010). Multiple adverbials in Chinese are more complex, and adverbials preceded by governor words often involve knowledge above the syntactic level (Jin, 2008; Guo, 2013). Thus, the typological characteristics of Chinese adverbials are more difficult to acquire than subjects, objects, and attributives in both the written and spoken modalities. In addition, oral learners are more likely to make errors in word order than writing learners, and they still make errors in word order in the final G7 stage, as shown in (23), where ECSL learners placed the adverb *yigong* (“in total”) after *shi* (“is”). As the language proficiency increases, ECSL learners in the written modality gradually break away from the negative transfer of their native language and make fewer errors in word order, which can be avoided at the high level, while ECSL learners in the spoken modality make more errors in word order, which are still present at the high level. This also confirms the previous hypothesis that ECSL learners in the spoken modality have more errors in word order and are more susceptible to the influence of their native language in the adverbial structures, which may be closely related to the greater time pressure and cognitive load in the spoken language.

(15) ^*^Tā **chūshēng** 1949nián. (O4, t70, s4)
  He **born** the *year* of *1949*
  “He **was born** in *1949*.”
  “Tā 1949nián **chūshēng**.”

(16) ^*^Wǒ bàba zhǐhǎo **dāngyàoqiánderén** zàimǎlùshàng. (W1, t478, s10)
  I dad had **to be beggar** on the road
  “My dad had **to be beggar** on the road.”
  “Wǒ bàba zhǐhǎo zàimǎlùshàng **dāngyàoqiánderén**.”

(17) ^*^Tā **fǎngwèn** wo zàiBólín. (O1, t81, s4)
  She **visit** me in Berlin.
  “She **visited** me in Berlin.”
  “Tā zàiBólín **fǎngwèn** wo.”

(18) ^*^Gēn biérén **jiāoliú** yòngkǒuyǔ. (O2, t200a2, s5)
  With others **communica**te in spoken language
  “**Communicating** with others in spoken language.”
  “Gēn biérén yòngkǒuyǔ**jiāoliú**.”

(19) ^*^Bùyòng **fèihěndàdexin** wèilezhǔnbèi. (W5, t710, s16)
  No need **take a lot of effort** to prepare
  “It doesn't **take a lot of effort** to prepare.”
  “Bùyòng wèilezhǔnbèi **fèihěndàdexin**.”

(20) ^*^Jiēshòushìjiè wèileàoyùnhuì. (O5, t166, s6)
  **Accept world** for the Olympics
  “**Accepting the world** for the Olympics.”
  “Wèileàoyùnhuì **jiēshòushìjiè**.”

(21) ^*^Wǒ hé tā **yǒu** chàbuduō yī nián le. (O3, t120, s6)
  I with he **have been** almost a year (AUX)
  “I **have been** with him for almost a year.”
  “Wǒ hé tā chàbuduō **yǒu** yī nián le.”

(22) ^*^Wǒ **qù** gēnwǒdetóngxué. (O1, t80, s2)
  I **go** with my classmates
  “I'm **going** with my classmates.”
  “Wǒ gēnwǒdetóngxué **qù**.”

(23) ^*^Zhège shíxí **shì** sāngè yuè yígòng.(O7, t213-7, s10)
  This internship **is three** month in total
  “This internship **is** three months in total.”
  “Zhège shíxí yígòng **shì** sāngè yuè.”

## Conclusions

Based on a syntactically-annotated corpus of Chinese interlanguage that we built, and dependency direction used as a metric, this paper reveals the development in the typological characteristics of Chinese interlanguage across both written and spoken language modalities. After the above discussion and analysis, we have answered the four questions raised at the beginning of this paper. The results show that there are differences in the typological characteristics of Chinese interlanguage in different modalities at varied L2 proficiency levels.

For Question 1, the current study demonstrates that dependency directions differed in both Chinese and English native languages under different modalities. The HF% of the spoken native Chinese modality was larger than that of the written modality; the HF% of the spoken native English modality was smaller than that of the written modality.

For Question 2 and Question 3, the results of the study showed that the development trend of interlanguage typological characteristics differed in different modalities. In the written modality, the HI dependency direction of ECSL learners changed significantly with L2 proficiency. However, in the spoken modality, ECSL learners' dependency direction was not related to L2 proficiency, probably due to the greater time constraint and cognitive load on speakers' language output, and the spoken output was more difficult for learners, which led to more errors in word order. In addition to this, the presence of syntactic component inversions in the target language (Chinese) spoken modality itself also affects the changes in dependency direction. Now we can answer Question 3, that dependency direction, as a proven measure of typological development in the written modality, does not effectively reflect interlanguage development in the spoken modality and does not seem to be a generic indicator of typology across modalities.

In terms of the acquisition of typological characteristics of important linguistic structures, ECSL learners in different modalities also showed different typological characteristics. Chinese and English are both SVO languages, so ECSL learners in both modalities can acquire subject-verb and verb-object structures in Chinese well, but the spoken modality was more prone to errors in word order and component inversions than the written modality. There is a difference in the attributive position between Chinese and English; the attributive structure in Chinese precedes the modified noun, while the position of the attributive structure in English is flexible and can either precedes or follows the modified noun. So it is relatively not difficult for ECSL learners to adjust the definite position. In the attributive structure, compared to the written modality, ECSL learners in the spoken modality are more likely to make errors in word order and to be affected by negative native language transfer. The adverbial position is complex in both Chinese and English, and the adverbial structure is not easily acquired by ECSL learners in both modalities. Compared to the previous language structures, ECSL learners in both modalities are more likely to be affected by negative native language transfer and to make errors in word order in linguistic typology characteristics. However, ECSL learners of the spoken modality were more likely to make errors in word order in the adverbial structure than in the written modality. By far, we have answered Question 4 from both macro and micro perspectives.

Based on the above findings, it can be found that ESCL learners always face more difficulties in their oral output, both at macro and micro levels, which may shed light on language teaching. At the macro level involving the syntactic overview of Chinese syntax, ESCL learners' changes in language proficiency in the spoken modality cannot be measured by using the indicator of dependency direction, and they are more prone to make errors in word orders at all stages. It can be seen that L2 learners' speaking performance is unstable and seems to be more susceptible to other factors. Therefore, speaking instruction should not be neglected at all language teaching stages. In addition to imparting knowledge, the focus should be on developing L2 learners' ability to apply their knowledge and integrating speaking training throughout the teaching process. In terms of specific language structures at the micro level, errors in word order are more likely to occur where there are differences between the native language and the target language, and the more flexible the positions of certain syntactic structures are, the more likely it is that the errors in word order will occur (i.e., the adverbial structure). Therefore, when teaching language structures, teachers should have a good idea of what to do and choose the appropriate teaching methods according to the difficulty of syntactic structures.

By virtue of the methodology widely used in quantitative linguistics, this paper reveals the overall typological characteristics of the interlanguage in terms of modality. The results show that the typological characteristics of ECSL learners' mediated speech differ across modalities, and that dependency direction, as a typological indicator proven to be valid in the written modality, does not seem to be applicable to the spoken modality. We adopted a new research paradigm to examine the typological characteristics of the whole interlanguage, which helps to broaden the scope of linguistic typology. Additionally, it also contributes to the application of linguistic research methods in the field of second language acquisition. Of course, our study of different modalities of interlanguage is only a preliminary attempt, and further research is needed to verify the findings. There are only a few studies on the typology of interlanguage using quantitative linguistics, and they merely involve English and Chinese as interlanguage. So studies on other languages as interlanguage are yet to be explored.

## Data availability statement

The raw data supporting the conclusions of this article will be made available by the authors, without undue reservation.

## Author contributions

YH and HL conceived and designed the study. YH and XX collected the data and performed the statistical analysis. All authors contributed in result interpretation and manuscript writing.

## Footnotes

^1^Linguistic typology covers syntactic, lexical and phonological universals. In the present study, the discussion only involves the syntactic level.

^2^It is important to note that most cross-modal studies usually consider spoken language and written language are of the same modality, i.e., “aural-oral modality,” which is used to distinguish from sign language (Namir and Schlesinger, 1978; Schwager and Zeshan, 2008). However, the different production patterns and cognitive processes of spoken and written languages do result in differences in syntax, for example, the difference in word order. Our study focuses on the similarities and differences in word order of spoken and written languages, but it does not mean that we consider spoken and written languages as those belonging to completely different modalities.

^3^This sentence is from O3, t203a, s20 (t203a:text of 203a of J4; s20: sentence20 in 203a).

^4^In order to distinguish it from the case of the wrong word order, a small dot is added under the part with inverted components.

^5^The correct word order in Chinese should be “Deguo (de) Bolin” (Berlin of Germany), and the “de” in the adverbial structure can be omitted here, it should be “Deguo Bolin”.
